# A genetic variant in *IL‐15R*α correlates with physical activity among European–American adults

**DOI:** 10.1002/mgg3.368

**Published:** 2018-04-06

**Authors:** Michael Bruneau, Sean Walsh, Eric Selinsky, Garrett Ash, Theodore J. Angelopoulos, Priscilla Clarkson, Paul Gordon, Niall Moyna, Paul Visich, Robert Zoeller, Paul Thompson, Heather Gordish‐Dressman, Eric Hoffman, Joseph Devaney, Linda S. Pescatello

**Affiliations:** ^1^ Drexel University Philadelphia PA USA; ^2^ Central Connecticut State University New Britain CT USA; ^3^ University of Connecticut Storrs CT USA; ^4^ Yale University New Haven CT USA; ^5^ Emory & Henry College Emory VA USA; ^6^ University of Massachusetts Amherst MA USA; ^7^ Baylor University Waco TX USA; ^8^ Dublin City University Dublin Ireland; ^9^ University of New England Biddeford ME USA; ^10^ Florida Atlantic University Boca Raton FL USA; ^11^ Hartford Hospital Hartford CT USA; ^12^ Children's National Medical Center Washington DC USA; ^13^ Cooperative International Neuromuscular Research Group Washington DC USA; ^14^ University of Connecticut Institute for Systems Genomics Storrs CT USA

**Keywords:** cytokine, exercise, polymorphisms

## Abstract

**Background:**

Interleukin‐15 (IL‐15) is a myokine associated with muscle strength, possibly by attenuating protein breakdown. A variant in the alpha‐receptor (*IL‐15R*α 1775 A>C, rs2228059) partially modulates the muscle strength and size response to resistance training. We examined if this polymorphism associated with habitual physical activity among European‐American adults.

**Methods:**

Men (*n *=* *240, 23.7 ± 0.3 year, body mass index [BMI] 25.3 ± 0.3 kg/m^2^) and women (*n *=* *292, 23.2 ± 0.3 year, 24.0 ± 0.3 kg/m^2^) were genotyped. Physical activity phenotypes were derived from the Paffenbarger Physical Activity Questionnaire. Analysis of covariance (ancova) tested log‐transformed differences between the *IL‐15R*α genotype and physical activity phenotypes by gender with age and BMI as covariates.

**Results:**

Men with the *IL‐15R*α 1775AA genotype spent more time in light intensity physical activity (39.4 ± 2.4 hr/week) than men with the CC genotype (28.6 ± 2.3 hr/week, (*p *=* *.009).

**Conclusion:**

Further research is needed to confirm our finding and determine the possible mechanisms by which the *IL‐15R*α variant modulates light intensity physical activity.

## 1 INTRODUCTION

A physically active lifestyle is vital to physical and mental well‐being (Haskell et al., 2007). Consequently, the American College of Sports Medicine recommends that most adults engage in moderate‐intensity aerobic exercise for ≥30 min/day on ≥5 day/week for a total of ≥150 min/week; vigorous‐intensity aerobic exercise for ≥20 min/day on ≥3 day/week (≥75 min/week); or a combination of these moderate‐ and vigorous‐intensity exercise recommendations to achieve a total energy expenditure of ≥500–1,000 MET‐min/week (American College of Sports, 2017; Garber et al., 2011). Despite awareness of the myriad of health benefits that result from habitual physical activity, only one in five adults in the United States meet these physical activity recommendations (Troiano et al., 2008). In addition, 25% of Americans do not participate in any leisure time physical activity (Centers for Disease Control and Prevention, 2013). Given these low rates of adherence to a physically active lifestyle, it is imperative to gain insight into factors that may modulate habitual physical activity so that effective interventions can be developed.

Physical activity has a significant genetic component as evident in twin studies with heritability estimates between 50%–90% (Joosen, Gielen, Vlietinck, & Westerterp, 2005; de Moor et al., 2007; Stubbe et al., 2006). “Activity genomics” is a growing field indicating that the genetic basis of physical activity is accounted for by the sum of small gene effects rather than a small number of genes with a large effect (de Moor et al., 2007, 2009). Furthermore, the genetic influence on habitual physical activity appears to be more important in young adulthood than adolescence (Stubbe, Boomsma, & de Geus, 2005; Vink et al., 2011).

A gene that is a logical candidate to explore regarding its effects on habitual physical activity is the interleukin‐15 alpha specific receptor (*IL‐15R*α) for the various reasons that follow. Signaling by IL‐15 occurs not only through binding with its trimeric receptor but also through a complex formed between IL‐15 and IL‐15Rα such that the biological activity of IL‐15 in vivo is likely regulated through its interactions with IL‐15Rα (Pistilli et al., 2011). IL‐15 is a myokine that inhibits cell death (i.e., anti‐apoptotic) and promotes the production of many immune cells including natural killer (NK) cells, cluster of differentiation 8 (CD8+) T cells, neutrophils, eosinophils, mast cells, monocytes, and B lymphocytes (Budagian, Bulanova, Paus, & Bulfone‐Paus, 2006).

In addition to its anti‐apoptotic action on immunological cells, IL‐15 plays a role in protein, lipid, and glucose metabolism. IL‐15 reduces fat mass in rodents (Barra et al., 2010; Carbo et al., 2001), and its effect on lipids in white adipose tissue may result via its mediation of calcineurin (Almendro et al., 2009), which decreases white adipose mass by affecting preadipocyte differentiation (Quinn, Strait‐Bodey, Anderson, Argiles, & Havel, 2005) and reducing lipogenesis (Carbo et al., 2001). Cell culture studies indicate IL‐15 also stimulates lipolysis (Ajuwon & Spurlock, 2004) and adiponectin secretion (Quinn et al., 2005). Lastly, IL‐15 regulates glucose metabolism in vitro (Busquets, Figueras, Almendro, Lopez‐Soriano, & Argiles, 2006), and recent work by He et al. (2010) and Wu, He, et al. (2010) has revealed differences in spontaneous cage activity when comparing *IL‐15R*α control mice and *IL‐15R*α knockout mice, whereby the knockout mice displayed hyperactivity in comparison to the control mice. Collectively, the regulatory actions of IL‐15Rα on inflammatory and metabolic processes on muscle and adipose tissue and *IL‐15R*α spontaneous cage activity of mice suggest they could be linked in some fashion.

Furthermore, *IL‐15R*α 1775A>C (rs2228059; OMIM *601070; GenBank NC_000010.11) has been reported to be associated with several health and fitness‐related phenotypes including adiposity, muscular size and strength, and lipid‐lipoproteins (Arnett et al., 2009; He et al., 2010; Pistilli et al., 2008; di Renzo et al., 2009; Riechman, Balasekaran, Roth, & Ferrell, 2004; Wu, He, et al. (2010)). Our research group from the Functional Single Nucleotide Polymorphisms Associated with Muscle Size and Strength (FAMuSS NIH R01 NS40606‐02) study found that *IL‐15R*α 1775 A>C modulated baseline whole muscle volume and quality and serum cholesterol among European‐American men and women from FAMuSS (Pistilli et al., 2008).

The strongest candidate genes are those whose association with the phenotype in question is supported by multiple lines of evidence (Dipetrillo, Wang, Stylianou, & Paigen, 2005; Lightfoot, 2011). In the case of habitual physical activity, such criteria include genetic variants reported to be associated with chronic diseases and health conditions related to physical inactivity (Arnett et al., 2009; Gokkusu et al., 2010; di Renzo et al., 2009), those with functional relevance to exercise (Riechman et al., 2004; Scanzello et al., 2009), and those previously reported to be associated with exercise performance and health and fitness‐related phenotypes (Bray et al., 2009; Pistilli et al., 2008). *IL15R*α 1775A>C meets these criteria. Therefore, the purpose of our study was to test the hypothesis that *IL‐15‐R*α 1775 A>C would influence habitual physical activity in a large subsample of 532 young European‐American adults from FAMuSS (Pescatello, Devaney, Hubal, Thompson, & Hoffman, 2013; Thompson et al., 2004).

## 2 METHODS

### 2.1 Ethical compliance

The FaMuSS study was conducted by the Exercise and Genetics Collaborative Research Group consisting of researchers from the University of Connecticut, Dublin City University, University of Massachusetts, Central Michigan University, University of Central Florida, Florida Atlantic University, West Virginia University, Yale University, Hartford Hospital, and the Children's National Medical Center. The institutional review boards from all 10 institutions approved the study protocol. The primary aim of FAMuSS was to identify genetic factors that dictated the response of health‐related fitness phenotypes to resistance training (RT). However, we took the opportunity to assess habitual physical activity with the Paffenbarger Physical Activity Questionnaire (PPAQ) (Paffenbarger, Wing, & Hyde, 1978) prior to RT. The experimental design of FAMuSS has been described in detail previously so only the methods related to this substudy are described (Clarkson et al., 2005; Kostek et al., 2007; Pescatello et al., 2006, 2013; Thompson et al., 2004).

### 2.2 Subjects

All subjects provided written informed consent. Potential study volunteers were excluded if they: (1) had performed RT in the past year; (2) had a chronic condition that would preclude their ability to perform RT; (3) had metal implants in the arms, eyes, head, brain, neck, and/or heart that would be contraindicated to magnetic resonance imaging (MRI); (4) were prescribed and/or taking medications (i.e., corticosteroids, anabolic steroids, antihypertensive or antilipidemic medications, diuretics, Depo‐Provera contraceptive injection, Clenbuterol, Rhinocort nasal inhaler, lithium nonsteroidal anti‐inflammatory medications) known to effect skeletal muscle function; (5) consumed an average of ≥ two alcoholic drinks per day; (6) consumed dietary supplements to enhance muscle strength and size or weight; or (7) gained or lost >2.2 kg within 3 months prior to enrolling in the study. In all, 532 European American adults (≥18 years) were genotyped for *IL‐15* A>C and comprised the sample of this substudy.

### 2.3 Physical activity

Upon entry into FAMuSS, subjects were measured for body weight (kg) and height (cm) to determine body mass index (BMI, kg/m^2^). Subjects then completed the PPAQ (Ainsworth, Leon, Richardson, Jacobs, & Paffenbarger, 1993; Paffenbarger et al., 1978). The PPAQ is an accurate and reliable measure of leisure time physical activity (Ainsworth et al., 1993; Paffenbarger, Blair, Lee, & Hyde, 1993; Paffenbarger et al., 1978; Simpson et al., 2015). The physical activity phenotypes examined in this substudy were derived from the following question: “On a usual weekday and weekend day over the past year, how much time do you spend in the following activities‐vigorous, moderate, and light intensity physical activity, sitting and sleeping?”. The self‐reported time in hours (hr) spent in vigorous, moderate, and light intensity physical activity and time spent sitting were totaled and used for comparison among the *IL‐15* A>C genotypes (Paffenbarger et al., 1993).

### 2.4 Genotyping

Fasting venous blood samples were taken from each subject in standard EDTA vacutainer tubes (Becton Dickson and Company, Franklin Lakes, NJ, USA). Samples remained anonymous and were sent to Children's National Medical Research Center in Washington, D.C for processing using the Gentra Puregene Blood DNA Purification kit (Qiagen, Valencia, CA, USA). Genotyping of the *IL‐15* A>C polymorphism was performed with TaqMan allele discrimination assays employing a 5′ nuclease activity of Taq polymerase detecting a fluorescent reporter signal generated during polymerase chain reactions (PCR) according to the manufacturer's protocol for a 10 μl reaction. The PCR profile was 10 min at 95°C (denaturation), 44 cycles of 15 s at 92°C, and 1 min at an annealing temperature of 60°C. All Taqman allelic discrimination reactions were analyzed using an ABI 7900 real‐time PCR system (Applied Biosystems, Foster City, CA, USA). For quality control, negative controls (water blanks) and duplicate samples covering 5% of the total number of samples analyzed (100% agreement) were included in the genotyping analysis.

### 2.5 Statistical analysis

Chi‐square analysis determined *IL‐15R*α 1775A>C was in Hardy‐Weinberg equilibrium (HWE) (*p *<* *.05). Analysis of covariance (ancova) was used to test for differences among *IL‐15R*α genotypes and physical activity phenotypes by sex with age and BMI as covariates. In this model, log‐transformation was performed for the physical activity phenotypes to satisfy the underlying assumption of normality. For ancova models reaching statistical significance, post‐hoc pair‐wise comparisons were performed between genotypes with Bonferroni adjustments to control for multiple comparisons. Because *IL‐15R*α genotype physical activity phenotype associations revealed differential patterns in men and women, our results are displayed for the total sample and by men and women, separately. The proportion of all variance in physical activity phenotypes attributable to *IL‐15R*α genotype was determined using the likelihood test ratio. Alpha levels were set a priori at *p *<* *.05, and all statistical analyses were performed using the Statistical Package for the Social Sciences (SPSS, Armonk, NY, USA) 24.0.

## 3 RESULTS

### 3.1 Subject characteristics

FAMuSS subjects (*n *=* *532) genotyped for the *IL‐15R*α (rs2228059) polymorphism consisted of 240 men and 292 women of European‐American decent (Table 1). Age did not differ between men and women (*p *≥* *.05); however, men had a higher BMI than women (*p *=* *.04).

**Table 1:** Subject characteristics (X ± *SEM*) for *IL‐15R*α A>C (rs2228059) among the FAMuSS subsample

Variable	Total sample (*n *=* *532)	Men (*n *=* *240)	Women (*n *=* *292)
Age (year)	23.4 ± 0.2	23.7 ± 0.3	23.2 ± 0.3
BMI (kg/m^2^)	24.6 ± 0.2	25.3 ± 0.3^a^	24.0 ± 0.3

FAMuSS, functional single nucleotide polymorphisms associate with muscle size and strength study.

a Statistically significant difference between men and women (*p *=* *.04).

### 3.2 IL‐15Rα 1775 A>C genotype physical activity phenotype associations by sex

**Figure 1:**
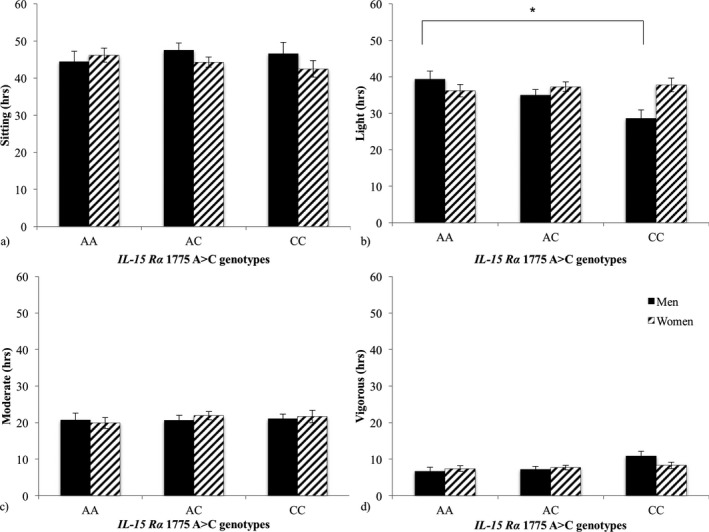
Time spent (X ± *SEM*) sitting (a) and in light (b), moderate (c), and vigorous (d) physical activity (hr) by *IL‐15R*α 1775 A>C (rs2228059) genotype and sex

A significant difference was observed amongst *IL‐15R*α 1775 A>C genotypes and light intensity physical activity levels (*F *=* *4.65, *p *=* *.01). Men with *IL‐15R*α 1775AA genotype spent more time in light intensity physical activity (39.4 ± 2.4 hr/week) than men with the CC genotype (28.6 ± 2.3 hr/week), (*p *=* *.009) explaining 5.8% of the variability in time spent in light intensity physical activity (Figure 1). However, time spent in sitting and moderate intensity physical activity did not differ by *IL‐15R*α 1775 A>C genotype among men. Furthermore, time spent in sitting and light, moderate, or vigorous intensity physical activity did not differ by *IL15R*α 1775 A>C genotype among women (*p *>* *.05) (Figure 1).

## 4 DISCUSSION

The aim of this substudy was to determine whether *IL‐15R*α 1775 A>C associated with habitual physical activity in a large subsample of healthy European‐American adults from FAMuSS (Pescatello et al., 2013; Thompson et al., 2004). The most noteworthy finding was the *IL‐15R*α 1775 A>C made a small but meaningful contribution to the interindividual differences we observed in habitual physical activity that varied by sex and the intensity of physical activity performed. Men homozygous for the A allele reported spending more time in light intensity physical activity than men homozygous for the C allele, explaining 5.8% of the variability in habitual physical activity; while these associations were not apparent in women. This finding is consistent with much of the work published by FAMuSS study investigators in that a persistent effect modifier of FAMuSS findings has been sex differences in the various phenotypes examined (Pescatello et al., 2013). Furthermore, they are consistent with the “Activity Genomic” literature that physical activity is a polygenetic trait such that there are many genes accounting for small effects (de Moor et al., 2007, 2009).

Previous work by He et al. (2010) using *IL15‐R*α knockout rodents revealed an association with locomotor activity, core temperature, and food intake. Similarly, Pistilli et al. (2011) used two different measures to quantify cage activity, cage wheel running and number of photobeam breaks during a 24 hr period. They observed a 6.3 fold increase in wheel revolutions during the 14 hr period with a 74% higher ambulatory count during the 24 hr collection period for *IL15‐R*α knockout mice compared to controls. These authors concluded that complete knockout of *‐IL15R*α resulted in a mouse with an increased capacity for activity.

Providing further evidence of *IL15‐R*α role in potentially mediating physical activity behavior, Pan et al. (2014) demonstrated that IL‐15 crosses the blood brain barrier, while work by Hsuchou, Pan, Wu, and Kastin (2009) and Hanisch et al. (1997) reported IL‐15 mRNA and its receptor subunits are also constitutively present in various brain regions. These findings indicate an active communication between blood‐borne IL‐15 and the central nervous system (He et al., 2010; Wu, Wang, Yeh, Lu, & Wu, 2010). Considering that biological activity of IL‐15 in vivo is likely regulated through its interactions with IL‐15Rα, the findings of Pistilli et al. (2011) in animals and now ours in humans suggest that differential receptor activity due to *IL‐15R*α genetic variation impacts physical activity levels in animals and humans.

There is no obvious explanation for the sex differences observed in the present substudy. However, previous candidate gene association studies involving health and fitness‐related phenotypes by our group have also observed sex‐specific differences (Arnett et al., 2009; Pistilli et al., 2008; di Renzo et al., 2009; Riechman et al., 2004). Although speculative, the sex differences that have been observed in several studies perhaps are partially due to sex‐specific hormonal differences. Work by Bowen, Turner, and Lightfoot (2011) using a rodent model suggests that physical activity in mice is affected by endogenous steroids and may be an important biological factor regulating physical activity. Therefore, due to the vastly different hormonal environments between men and women, it is unlikely that genetic variation influencing our physical activity phenotypes would be identical between sexes.

The public health significance of our findings is of importance considering genetic factors influencing physical activity levels are now viewed as “the core” of the transdisciplinary model of exercise behavior (Bryan et al., 2011). In this model, Bryan et al. (2011) stated that approaches in treating physical inactivity should emphasize elucidating relationships among genetic, physiological, and psychological variables that will aid in our understanding of individual differences in the initiation and maintenance of physical activity behavior (Bryan et al., 2011). Work in “Activity Genomics” may therefore provide an exciting new avenue that may help us better understand individual differences in the initiation and maintenance of physical activity behavior due to genetic predispositions eventually leading to the development of effective interventions to increase physical activity and decrease morbidity and mortality due to sedentary lifestyles (Bryan et al., 2011). Indeed, men with *IL‐15R*α 1775AA genotype spent more time in light intensity physical activity (39.4 ± 2.4 hr/week) than men with the CC genotype (28.6 ± 2.3 hr/week), which equates to a potential body weight differential of ~21.9 kg annually.

Similarly, Bann et al. (2015) evaluated the association between light intensity physical activity and BMI and found that a 1 hr/day increase in light physical activity was associated with a 0.46 kg/m^2^ reduction in BMI. Excessive body weight is a risk factor for numerous chronic diseases and health conditions including cardiovascular disease, diabetes mellitus, and several forms of cancer, among others (Benjamin et al., 2017). From a public health perspective, the genotype differences in light intensity physical activity we found of 10.8 hr/week, have the potential to minimize weight gain as well as lower the adverse health consequences of overweight and obesity.

This FaMUss substudy is not without limitation. Because the primary purpose of FaMuSS was to examine the influence of genetic variation on human muscle size and strength in response to RT, this study was not designed to examine habitual physical activity as a primary outcome measure. We assessed physical activity with a valid and reliable questionnaire (Paffenbarger et al., 1978) on populations with similar physical characteristics as ours to minimize the known limits of self‐report methods. However, the self‐reported recall of habitual physical activity from the previous year may have been limited by inaccuracies in subject reporting and/or social desirability bias (Chastin, Culhane, & Dall, 2014; Paulhus, 1991). We also did not measure biomarkers along the interleukin‐15 and its alpha specific receptor pathway, so further research is warranted to elucidate mechanisms for the sex and intensity dependent associations we observed. Nonetheless, the strengths of our substudy include the large sample of 532 healthy European American adults that was sufficiently powered to detect our observed effect size of *f *=* *0.19 with 98% power at an alpha set to *p *<* *.05 (Faul, Erdfelder, Lang, & Buchner, 2007). Our substudy also controlled for the potential confounding variables of age and BMI, which could have influenced the associations found between *IL‐15R*α and light physical activity.

## 5 CONCLUSION

In conclusion, the present results add a variant within *IL‐15R*α to a growing list of polymorphisms that have been identified as making contributions to the inter‐individual variation in habitual physical activity levels of humans. Further exploration into the *IL‐15R*α and other gene variants associated with physical activity should continue so that we better understand the role of genetic variation and its influence on physical activity.

## CONFLICT OF INTEREST

The authors declare no conflict of interest with this manuscript.
